# The changing pattern of bacterial and fungal respiratory isolates in patients with and without COVID-19 admitted to intensive care unit

**DOI:** 10.1186/s12879-022-07176-x

**Published:** 2022-02-23

**Authors:** Gianluca Zuglian, Diego Ripamonti, Alessandra Tebaldi, Marina Cuntrò, Ivano Riva, Claudio Farina, Marco Rizzi

**Affiliations:** 1 Infectious Diseases Unit, ASST “Papa Giovanni XXIII”, Piazza OMS, 1, 24127 Bergamo, Italy; 2 Microbiology and Virology Laboratory, ASST “Papa Giovanni XXIII”, Bergamo, Italy; 3 Intensive Care Unit, ASST “Papa Giovanni XXIII”, Bergamo, Italy

## Abstract

**Objectives:**

Severe acute respiratory syndrome 2 (SARS-CoV-2) pandemic has had a heavy impact on national health system, especially in the first wave. That impact hit principally the intensive care units (ICUs). The large number of patients requiring hospitalization in ICUs lead to a complete upheaval of intensive wards. The increase in bed, the fewer number of nurses per patient, the constant use of personal protective equipment, the new antimicrobial surveillance protocols could have had deeply effects on microbiological flora of these wards. Moreover, the overconsumption of antimicrobial therapy in COVID-19 patients, like several studies report, could have impact of this aspect. Aim of this study is to evaluate the changing pattern of microbiological respiratory isolates during and before COVID-19 pandemic in a tertiary hospital ICUs.

**Methods:**

A retrospective, observational study was conducted in ICUs of “ASST Papa Giovanni XXIII”, a large tertiary referral hospital in Northern Italy. We have retrospectively collected the microbiological data from bronchoalveolar lavage (BAL) and tracheal aspirate (TA) of patients with COVID-19, hospitalized in ICUs from 22nd February 2020 to 31st May 2020 (Period 1), and without COVID-19, from 22nd February 2019 to 31st May 2019 (Period 2). We compared the prevalence and the antibiotic profile of bacterial and fungal species in the two time periods.

**Results:**

The prevalence of *Pseudomonas* spp. shows a statistically significant increase from patients without COVID-19 compared to COVID-19 positive as well as the prevalence of *Enterococcus* spp. On the contrary, the prevalence of Gram negative non fermenting bacteria (GN-NFB), *Haemophilus influenzae* and *Streptococcus pneumoniae* showed a significant reduction between two periods. There was a statistically significant increase in resistance of *Pseudomonas* spp. to carbapenems and piperacillin/tazobactam and *Enterobacterales* spp. for piperacillin/tazobactam, in COVID-19 positive patients compared to patients without COVID-19. We did not observe significant changing in fungal respiratory isolates.

**Conclusions:**

A changing pattern in prevalence and resistance profiles of bacterial and fungal species was observed during COVID-19 pandemic.

## Introduction

Severe acute respiratory syndrome 2 (SARS-CoV-2) has spread worldwide since 2019. Patients with SARS-CoV-2 infections may develop a severe form of coronavirus disease (COVID-19) requiring hospitalization and admission to intensive care units (ICU) in approximately 30% of them [1, 2]. A large proportion of COVID-19 patients received antibiotic and antifungal therapy for proven or suspected co-bacterial infections during their ICU stay [3] and several studies have highlighted the antibiotic over-exposure in this population, despite the low rate of culture-proven bacterial co-infections [4]. This may be caused by several factors including the possible rapid evolution of COVID-19 to multi organ failure and worse outcome [5], the uncertainties about this new disease and the limitations of invasive diagnostic procedures due to the SARS-Cov-2 transmission precautions. Moreover, COVID-19 pandemic has represented an exceptional stress for the hospital setting, especially for the ICU [6, 7], due to the overwhelming number of patients requiring a prolonged ICU stay.

The above-mentioned factors may have affected the local fungal and bacterial epidemiology. The aim of this study is to describe the prevalence of bacterial and fungal species in a cohort of COVID-19 patients admitted to ICUs compared to patients without COVID-19 observed at the same hospital during the previous year.

## Materials and methods

### Study design and obtained data

A retrospective observational study was conducted at the “ASST Papa Giovanni XXIII”, a large tertiary referral hospital (990 beds) placed in Bergamo, one of the most affected provinces during the COVID-19 pandemic in Northern Italy in 2020. We identified two study periods: the first from 22nd February 2020 to 31st May 2020 (Period 1) and the second from 22nd February 2019 to 31st May 2019 (Period 2). We recruited all patients admitted to ICU wards with a BAL and/or TA performed in the two study periods. For period one, we recruited only patients with also a nasopharyngeal swab and/or BAL positive for SARS-CoV-2 before or until 48 h from admission to ICU wards. So, we identified two populations: the cohort of patients from period 1 (COVID-19 positive) and the cohort of patients from period 2 (COVID-19 negative). The requirement for written informed consent was waived due to the retrospective study design. The patient data obtained from the medical records included age, sex, comorbidities, causes of ICU admission, mechanical ventilation, hemodialysis, antibiotic/antifungal therapy and immunosuppression treatment (steroids/monoclonal antibodies) during ICU staying and outcome (death or discharged from ICU). Then, we compared the prevalence of bacterial and fungal species on the total of positive respiratory samples in the two time periods. TA and BAL were considered equally in the final analysis. The most prevalent bacterial species potentially pathogenic of the respiratory tract (*Enterobacterales*, *Pseudomonas* spp. and *Staphylococcus aureus*) were categorized on their antibiotic resistant profile as multidrug resistant (MDR) for *Enterobacterales* and *Pseudomonas* spp., (according to definitions by Magiorakos et al. [8].) and methicillin resistant (MR) for *Staphylococcus aureus* (according to cefoxitin screening).

### Respiratory samples protocol

In COVID-19 patients, a TA sampling was systematically performed every week during ICU staying. For every TA sample bacterial and fungal standard culture were requested. Moreover, BAL was performed, according to clinician’s judgment, when patient clinical condition required further diagnostic investigation due to the severity and possible differential diagnosis of respiratory impairment. In non-COVID-19 patients, TA and/or BAL were done when required.

### Statistical analysis

Categorical variables were reported using numbers and percentages, while continuous variables were reported using mean with standard deviation (SD). The categorical variables were analyzed using the χ^2^ test or Fisher’s exact test for small samples and we calculated the odds ratio (OD) whit 95% confidence interval (CI). All the statistical analyses were performed using JMP 16.0 (SAS Institute Inc., Cary, NC, USA). All the *P* values were two-sided, and statistical significance was set at *P* < 0.05.

### Ethics

All methods were carried out in accordance with Declaration of Helsinki. This study was approved by the ethics committee of The Papa Giovanni XXIII Hospital (Protocol N. 257/2020). To maintain the principle of confidentiality, the data used were anonymized. The need for informed consent was waived by the ethical Committee of the “Papa Giovanni XXIII” Hospital due to retrospective nature of the study.

## Results

A total of 194 patients were admitted to ICU with COVID-19 in period 1 (namely 65% of 297 patients admitted in ICU in that period), compared to 176 patients who were admitted in period 2. A total of 736 respiratory samples (3.8 per patient) and 392 (2.2 per patient) were collected in COVID-19 positive and negative patients, respectively. The proportion of positive respiratory specimens (for at least one pathogen, either bacterial or fungal) was 48% (355/736 samples) and 47.7% (187/392 samples) in COVID-19 positive and negative patients, respectively.

In Table 1, we reported the clinical characteristics of the two cohort of patients. COVID-19 patients were affected by more chronic heart diseases and obesity. During ICU stay, more patients were mechanically ventilated, underwent hemodialysis and received immunosuppression and antibiotic therapy.

**Table 1:** Clinical characteristics of patient with and without COVID-19

Patients characteristics	Overall	COVID-19	Non COVID-19	*p *value
Number	370	194	176	
Age, media (m-M)	59 (18–87)	60 (20–83)	57 (18–87)	0.09
Gender				
Male, N (%)	277 (74.9)	153 (78.9)	124 (70.5)	0.63
Female, N (%)	93 (25.1)	41 (21.1)	52 (29.6)	0.63
Time from hospital admission to ICU	6 (± 20)	4 (± 10)	8 (± 23)	0.1
Comorbidities				
Chronic heart disease, N (%)	99 (26.8)	**32 (16.5)**	**67 (38.7)**	** < 0.001**
Hypertension, N (%)	166 (44.9)	93 (48)	73 (41.5)	0.25
CKD, N (%)	21 (5.7)	7 (3.6)	14 (8)	0.11
Chronic liver disease, N (%)	28 (7.6)	**6 (3.1)**	**22 (12.5)**	**0.006**
Chronic neurological disease, N (%)	39 (10.5)	**11 (5.7)**	**28 (15.9)**	**0.002**
Cancer, N (%)	26 (7.0)	10 (5.2)	16 (9.1)	0.2
Immunodefic/hematological/SOT, N (%)	38 (10.3)	22 (11.3)	16 (9.1)	0.59
Lung disease, N (%)	44 (11.9)	18 (9.3)	26 (14.8)	0.14
DM, N (%)	67 (18.1)	41 (21.1)	26 (14.8)	0.15
Obesity, N (%)	61 (16.5)	**44 (22.7)**	**17 (9.7)**	**0.001**
Therapy during ICU stay				
Mechanical ventilation, N (%)	345 (93.2)	**187 (96.4)**	**158 (89.8)**	**0.02**
Haemodyalisis, N (%)	54 (14.6)	**39 (20.1)**	**15 (8.5)**	**0.003**
Immunosoppressive therapy, N (%)	142 (38.4)	**120 (61.9)**	**22 (12.5)**	** < 0.001**
Antibiotics/antifungals, N (%)	320 (86.5)	172 (88.7)	148 (84.1)	0.26
LOS in ICU, media (SD)	23 (± 24)	22 (± 20)	25 (± 27)	0.24
Outcome				
Death in ICU, N (%)	138 (37.3)	69 (35.6)	69 (39.2)	0.54

Differences statistically significant are given in bold

*ICU* intensive care unit, *CKD* chronic kidney diseases, *SOT* solid organ transplantation, *LOS* length of stay, *SD* standard deviation

Table 2 shows the prevalence of bacterial and fungal species by group. In both periods, the most frequent bacterial isolates were *Pseudomonas* spp. and *Enterobacterales;* The prevalence of *Pseudomonas* spp. shows a statistically significant increase from period 2 to period 1, as well as the prevalence of *Enterococcus* spp. On the contrary, the prevalence of Gram negative non fermenting bacteria (GN-NFB), *Haemophilus influenzae* and *Streptococcus pneumoniae* showed a significant reduction from in COVID-19 patients.

**Table 2:** Prevalence of fungal and bacterial pathogens from respiratory samples

	COVID-19	Non COVID-19	p value	OR	95% CI
*CoNS,* N (%)	10 (2.3)	5 (1.7)	0.75	1.38	0.47–4.09
*Enterobacterales,* N (%)	**101 (22.9)**	**102 (33.7)**	**0.002**	**0.59**	**0.42–0.81**
*Enterococcus* spp., N (%)	**35 (7.9)**	**7 (2.3)**	**0.002**	**3.65**	**1.6–8.32**
GN-NFB, N (%)	**12 (2.7)**	**19 (6.3)**	**0.03**	**0.42**	**0.2–0.88**
*Haemophilus* spp., N (%)	**1 (0.2)**	**11 (3.6)**	** < 0.001**	**0.06**	**0.01–0.47**
*Pseudomonas* spp., N (%)	**114 (25.9)**	**40 (13.2)**	** < 0.001**	**2.29**	**1.54–3.4**
*Staphylococcus aureus*, N (%)	50 (11.3)	37 (12.2)	0.8	0.92	0.59–1.45
*Streptococcus* spp., N (%)	**3 (0.7)**	**9 (3.0)**	**0.03**	**0.22**	**0.06–0.83**
Other pathogens, N (%)	8 (1.8)	8 (2.6)	0.61	0.68	0.26–1.89
*Aspergillus* spp., N (%)	48 (10.9)	21 (6.9)	0.09	1.64	0.96–2.8
*Candida* spp., N (%)	59 (13.4)	44 (14.5)	0.13	0.73	0.6–1.39
Total	441	303	–	–	–

Differences statistically significant are given in bold

*CoNS* coagulase negative staphylococci, *GN-NFB* gram negative non fermenting bacteria

Table 3 compares the prevalence of resistant pathogens and the resistance profile for the antibiotic of interest. There were no statistically significant differences between the proportion of resistant pathogens in the two study periods. Nevertheless, there was a statistically significant increase in resistance of *Pseudomonas* spp. to carbapenems and piperacillin/tazobactam and *Enterobacterales* spp. for piperacillin/tazobactam, in COVID-19 patients compared to non-COVID-19 patients.

**Table 3:** Prevalence of antibiotic resistant pathogens according to the antibiotic of interest

	COVID-19	Non COVID-19	p value	OR	95% CI
*Pseudomonas* spp. MDR, N (%)	42 (36.8)	14 (35)	0.83	1.08	0.51–2.3
*Pseudomonas* spp. PIP/TZ-R, N (%)	**75 (65.8)**	**15 (37.5)**	**0.001**	**3.21**	**1.52–6.77**
*Pseudomonas* spp. CARBA-R, N (%)	**60 (52.6)**	**10 (25)**	**0.005**	**3.33**	**1.49–7.45**
*Pseudomonas* spp. CTZ/CEF-R, N (%)	40 (35.1)	14 (35)	0.86	1	0.47–2.14
*Enterobacterales* MDR, N (%)	33 (32.7)	34 (33.3)	0.96	0.97	0.54–1.74
*Enterobacterales* PIP/TZ-R, N (%)	**33 (327)**	**18 (17.6)**	**0.02**	**2.27**	**1.17–4.37**
*Enterobacterales* CARBA-R, N (%)	0 (0)	0 (0)	–	–	–
*Enterobacterales* 3GC-R. N (%)	16 (15.8)	18 (17.6)	0.1	1.19	0.6–2.36

Differences statistically significant are given in bold

*MDR* multi drug resistant, *PIP/TZ-R/S* piperacillin/tazobactam resistant/susceptible, *CARBA-R/S* carbapenem resistant/susceptible, *CTZ/CEF-R/S* ceftazidime/cefepime resistant/susceptible

## Discussion

We observed a variation of microbiological respiratory isolates before and during COVID-19 pandemic. In non-COVID-19 patients, the prevalence of potentially pathogenic bacterial isolates from respiratory samples in ICU patients was aligned with the one of previous studies in the same settings [9]. In COVID-19 patients, we observed the reduction of several bacterial species, especially *Enterobacterales* and the parallel increase of *Pseudomonas* spp. and *Enterococcus* spp.

In the interpretation of this changing epidemiology, some observations may be useful.

Firstly, the isolation of bacterial and fungal species does not necessary imply an active infection caused by these pathogens and, certainly, the systematic respiratory tract sampling, aimed to early intercept an infectious complication in the context of the SAR-CoV-2 pneumonia [10], may have led to an overestimation of microbiological events compared to a standard and less aggressive approach.

Secondly, about the changing epidemiology for *Enterococcus* spp. and *Pseudomonas* spp., we expected that the longer ICU stay for COVID-19 patients compared to the ones hospitalized for other causes [11] represents an important risk factor for colonization and/or infection caused by these bacteria^.^ We did not observe this longer hospitalization for COVID-19 patients. So, we postulated that the heavily impact of COVID-19 pandemic in ICUs has led the basic infection control practices difficult to be followed [12].

Thirdly, the tropism for respiratory tract by *Pseudomonas* spp. is well known, especially in patients with underlying lung disease (such as cystic fibrosis and chronic obstructive pulmonary diseases [13]). In a recent surveillance of VAP in COVID-19 patients, *Pseudomonas aeruginosa* was the most common pathogen responsible for ventilator‐associated lower respiratory tract infections [14] and the most common isolate in a population of critically ill patients hospitalized for influenza-associated ARDS [15]. We can speculate that the combination of the lung impairment by SARS-CoV-2 and the predisposition of *Pseudomonas* spp. could act synergistically to put these patients at risk for colonization/infection by this pathogen.

Fourthly, most COVID-19 patients during ICU hospitalization received empirical antibiotic therapy [3]. In the ICU departments the antibiotics belonging to beta lactams class are the most widely used [15]. In our study, we observed a high resistance rate for this antibiotic class by *Pseudomonas* spp. and *Enterobacterales* in non COVID-19 patients, that was dramatically increased. We can speculate that the antibiotic pressure may have favored the emergence of resistant bacteria [16].

Fifthly, the decrease of other bacterial species associated to respiratory tract infections is consistent with the low incidence rate of co-bacterial infections in COVID-19 patient [17] and the global reduction of prevalence of other respiratory pathogens, secondary to the public health measures against COVID-19 [18].

Sixthly, the high degree of immunosuppression induced by steroid therapy, used as salvage therapy in COVID-19 patients during ICU stay in the first wave, may have influenced the rate and the microbiological pattern of respiratory bacterial/fungal complications compared to non-COVID-19 patients [19].

Our work has several limitations. First, as mentioned above, the clinical significance of the colonization of the respiratory tract has not been investigated as the aim of the study was to describe the changing microbiological scenario during the COVID-19 pandemic which may help designing antimicrobial stewardship programs. Second, the clinical characteristics of patients admitted to ICU were not deeply investigated, for example we reported just if the patients received antibiotic or fungal therapy, not for how long or witch types of molecules. Third, we focused only on the first wave of COVID-19 pandemic, to better characterize the impact of exceptional number of patients not expecting to need hospitalization in ICUs.

## Data Availability

The data sets used and analyzed during the current study are not publicly available due to privacy of the included patients but are available from the corresponding author [G.Z.] on reasonable request.
